# Ocular immune privilege in action: the living eye imposes unique regulatory and anergic gene signatures on uveitogenic T cells

**DOI:** 10.1101/2025.03.01.640701

**Published:** 2025-03-06

**Authors:** Zixuan Peng, Vijayaraj Nagarajan, Reiko Horai, Yingyos Jittayasothorn, Mary J. Mattapallil, Rachel R. Caspi

**Affiliations:** 1 Laboratory of Immunology, National Eye Institute, National Institutes of Health, Bethesda, MD, USA; 2 Department of Ophthalmology, Xiangya Hospital, Central South University, Changsha, China

**Keywords:** Ocular immune privilege, autoimmune uveitis, Tregs, T cell anergy, single-cell transcriptomics, immune tolerance

## Abstract

Despite ocular immune privilege, circulating retina-specific T cells can trigger autoimmune uveitis, yet intraocular bleeding—a relatively common event—rarely leads to disease. Using an in vivo immune privilege model, we previously reported that all naïve retina-specific T cells entering the eye become primed *in situ*; about 30% become Foxp3+ T-regulatory cells (Tregs), while the rest fail to induce pathology. Here, single-cell transcriptomics and functional validation revealed distinct phenotypes in both populations: ocular Tregs were highly suppressive, whereas non-Tregs expressed suppression- and anergy-associated genes and lacked regulatory function. Trajectory analyses suggested that Tregs and anergic cells arise from a common proliferative precursor in parallel, rather than sequentially. Our data indicate a key checkpoint governing the divergence of anergic and regulatory fates. These findings provide molecular-level insights into ocular immune privilege and may inform strategies to silence autoimmune effector cells or reverse T cell unresponsiveness in cancer, vaccination, or chronic infection.

## Graphical Abstract

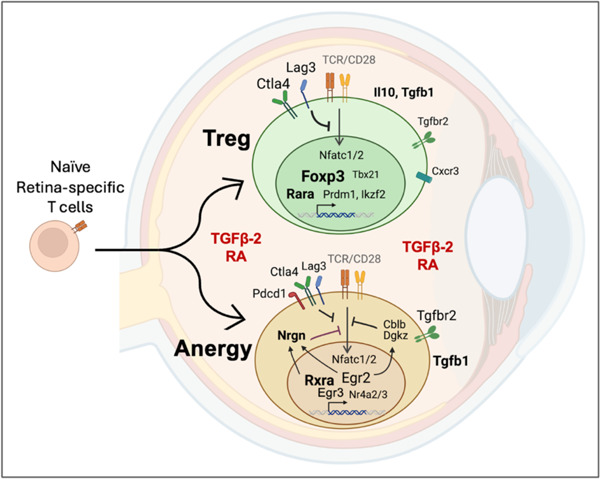

## INTRODUCTION

The eye has developed evolutionary adaptations that limit local inflammation in order to protect vision, that collectively form the complex phenomenon known as ocular immune privilege ^1 2^. In addition to the physical blood-tissue barriers that separate the eye from the immune system, multiple studies described that the intraocular environment, composed of ocular fluids and ocular resident cells, is immunosuppressive and can inhibit the activity of immunocompetent cells ^3 4 5^. Aqueous humor (AH) has been shown to reduce proinflammatory cytokine production by T cells in culture and to promote induction of regulatory T cells (Tregs) ^6 7^. Soluble factors involved in these processes include transforming growth factor-beta (TGF-β), α-melanocyte-stimulating hormone (α-MSH), vasoactive intestinal peptide (VIP), retinoic acid (RA), and others ^5 2^. Retinal glial Müller cells were the first ocular resident cells shown to inhibit T cells in co-culture ^8^. Since then, many reports described induction of Tregs by pigmented epithelia in the front and back of the eye ^3 9^. Immunomodulatory molecules expressed by these cells include CD86 ^10^ and PD-L1 ^11^ that engage the inhibitory receptors CTLA-4 and PD-1 on T cells, respectively, as well as membrane-bound or soluble TGF-β and CTLA-2α ^12^. Retinal microglia and dendritic-like cells also have been reported to inhibit antigen-specific T cell responses and to induce Tregs, possibly through aberrant antigen presentation ^13 14 15^. However, these studies were conducted largely *in vitro*, and could not represent the complexity of the living eye. Further, many predated the discovery of Forkhead box P3 (Foxp3) as a marker for Tregs, making it difficult to distinguish *de novo* induction of Tregs, from expansion of a preexisting Treg population.

Immune sequestration of unique retinal antigens (Ag), which are absent in the periphery, behind a blood-retinal barrier impedes development of peripheral tolerance in autoreactive T cells that escaped thymic negative selection ^2 16^. Such cells can be easily triggered to become pathogenic effectors, but nevertheless, autoimmune uveitis remains a relatively rare disease ^17^. To address the question how the eye maintains immune homeostasis, we established a mouse model in which retina-specific T cells, capable of inducing autoimmune uveitis, are injected into the eye ^18^. This model exposes the eye to naïve but non-tolerant T cells, as would occur in case of intraocular bleeding as a result of trauma or vascular abnormalities (e.g., macular degeneration, diabetic retinopathy, or neovascular glaucoma) ^19 20^. Interestingly, the T cells acquired an antigen-experienced (primed) phenotype within the eye but failed to induce uveitis. While ~30% converted to Foxp3^+^ Tregs, the majority did not, and produced detectable levels of IFN-γ and IL-17A ^18^. However, the fate and function of these eye-primed cells could not be determined, due to lack of appropriate technology.

In the current study, we utilized single-cell RNA sequencing (scRNA-seq) to comprehensively define the transcriptome of retina-specific T cells responding to their cognate antigen in the privileged intraocular environment. We present evidence that the non-Foxp3 converted population is not effectors being kept in check by the Tregs, but rather represents a novel anergic phenotype unique to the eye that differentiates in parallel with Foxp3^+^ Tregs from naive retina-specific T cells. Our findings are the first to dissect the phenomenon of ocular immune privilege at the molecular level.

## RESULTS

### Naive retina-specific T cells differentiate into several distinct subtypes within the ocular environment

**Fig. 1:**
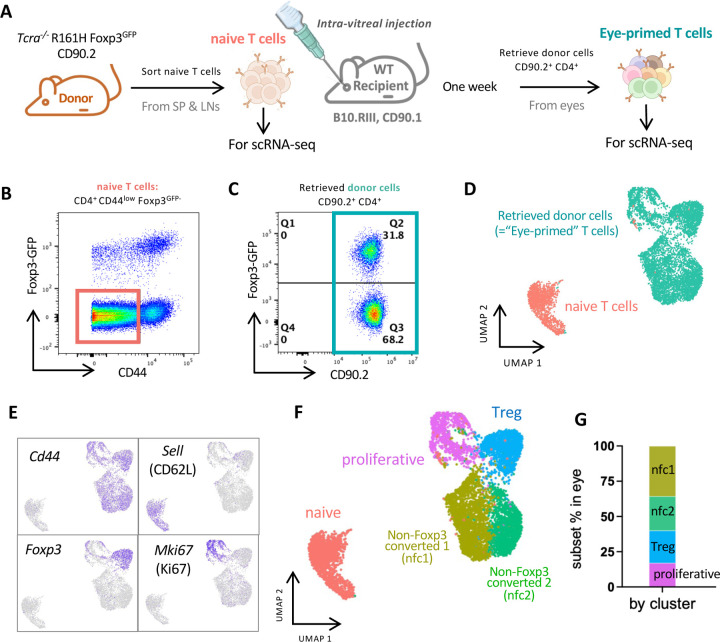
Naive retina-specific T cells differentiate into several distinct subtypes within the ocular environment (A) Retina-specific naive T cells were sorted from peripheral lymphoid tissues (spleens and lymph nodes) of Tcra−/− R161H Foxp3GFP reporter donor mice and injected into the eyes of CD90.1 congenic WT recipients. One week later, the CD90.2 donor T cells (eye-primed T cells) were retrieved from both eyes of 4 recipients. Naive and eye-primed T cells were subjected to scRNA-seq. (B) Gating strategy for the naive donor cell sorting (CD4+CD44lowFoxp3GFP-). (C) CD90.2+ CD4+ T cells retrieved from eyes of recipients were analyzed for the Foxp3 expression by flow cytometry. (D) UMAP showing naive T cells and eye-primed T cells. (E) Feature plots showing expression of antigen-primed T cell phenotype (CD44+ CD62L-), Treg maker (Foxp3) and proliferation marker (Mki67) in eye-primed T cells indicated by purple color. (F) Cluster identification and (G) Ratios of T cell subtypes of eye-primed T cells.

To gain better insight into the transcriptomic landscape that naive retina-specific T cells acquire within the eye, we performed scRNA-seq using the *in vivo* ocular immune privilege model. Briefly, naïve T cells were fluorescence-activated cell sorting (FACS)-sorted from *Tcra*^−/−^ R161H Foxp3^GFP^ CD90.2 mice and were intravitreally injected into the eyes of WT CD90.1 congenic recipients (Fig. 1A). The gating strategy for obtaining naive retina-specific non-Treg T cells from donors is depicted in Fig. 1B. One week after the injection, CD4^+^ CD90.2^+^ donor-derived T cells were retrieved from the eyes of CD90.1 congenic recipients. As we reported previously, about a third (31.8%) of the cells converted to Foxp3^+^ phenotype (Fig. 1C), and represent functionally competent Tregs ^18^. The naive retina-specific T cells before intravitreal injection and the injected cells retrieved from the recipient’s eyes were labeled by hashtag oligos before pooling and subjected to scRNA-seq (Fig. 1A and Table S1).

Using standard scRNA-seq analysis pipelines ^21^, cells that passed quality control were used for downstream analyses. Cells before- and after-injection into the eyes showed a clear division in the UMAP (Fig. 1D). A distinct pattern of high *Cd44* and almost no CD62L (*Sell*) expression was confirmed in all cells recovered from the eyes, indicating that they had been primed (Fig. 1E). The great majority of *Foxp3*^+^ Tregs formed one separate cluster, while a few *Foxp3*^*+*^ Tregs were detected in the neighboring cluster with high levels of the proliferation marker *Mki67* (Fig. 1E). This was in line with our previous finding that acquisition of Foxp3 expression in the ocular environment was accompanied by proliferation ^18^. Unsupervised clustering further uncovered five major populations (Fig. 1F) based on their differentially expressed genes (Figure S1A). Among them, naive (*Foxp1, Lef1, Satb1*), Treg (*Foxp3* and *Il10*), and proliferative (*Mki67, Stmn1*, *Top2a*) clusters were straightforward to annotate (Fig. 1E and Figure S1A). However, we did not find a clear pattern of gene expression to help classify the other two *Foxp3*-negative clusters as known lineages (Figure S1A). Therefore, for lack of a better definition, they are designated as “non-Foxp3 converted clusters” (nfc1 and nfc2) in the interim. The proportions of the T cell subpopulations recovered from recipients’ eyes are shown in Fig. 1G). Taken together, the scRNA-seq data revealed that the naive T cells exposed to their cognate antigen within the ocular environment differentiated from a homogeneous population into diverse subtypes, with Treg cells constituting one of several discrete populations.

### Non-Foxp3-converted (nfc) clusters do not conform to canonical gene patterns of known effector Th-lineages

To assess the phenotype of the non-Foxp3 converted (nfc) cells, we screened defining gene sets, including master transcription factors (TFs), signature cytokines, chemokines and surface molecules characteristic of the known major T-effector lineages.

**Fig. 2:**
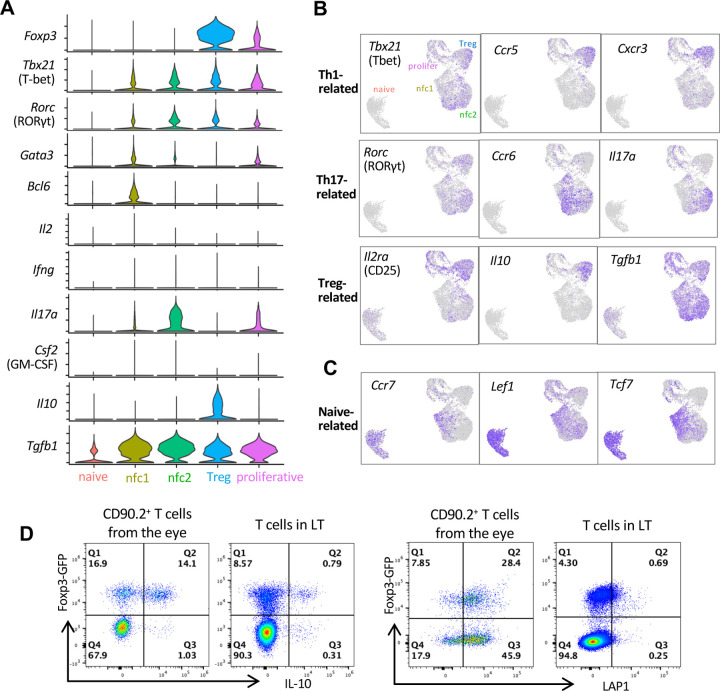
Non-Foxp3-converted (nfc) clusters do not conform to canonical gene patterns of effector Th-lineages (A) Violin plots showing the frequency of cells expressing Th lineage-specific transcription factors (TFs) and cytokines in each cluster. (B) Feature plots showing the distribution of selected Th lineage-specific markers including TFs, cytokines and chemokine receptors. (C) Feature plots showing distribution of markers associated with a naïve-like phenotype (Ccr7, Lef1, Tcf7). (D) FACS plots showing LAP-1 (encoded by the Tgfb1 gene) and IL-10 expression in Foxp3-converted vs. non-Foxp3-converted recovered from eyes of recipient mice after 1 week. LT=lymphoid tissue T cells from naïve R161H donor mice.

Levels of Th1 and Th17 lineage-defining transcription factors, *Tbx21* for Th1 and *Rorc* for Th17, respectively, were relatively low across all four eye-primed clusters, and not restricted to any particular cluster (Fig. 2A–B). Th1-associated surface markers (*Ccr5* and *Cxcr3)* ^22^, were confined to the Treg cluster, but absent in the nfc clusters (Fig. 2B). Additionally, *Ifng, and Csf2*, key pro-inflammatory Th1 cytokines, were undetectable in both nfc clusters (Fig. 2A), suggesting there is no Th1 induction. Although a moderate *Il17a* was present in nfc2 cluster (Fig. 2A), other Th17 signature genes (*Csf2, Il17f, Il22*, and *Il23r)* ^23,24^ were not detected (Fig. 2A and Figure S1B). The Th17 surface marker *Ccr6* was also not prominently expressed in nfc2 (Fig. 2B), making it inconsistent with a canonical Th17 profile. The nfc1 cluster exhibited modest expression of TF *Gata3* for Th2 and *Bcl6* for T follicular helper (Tfh) cells (Fig. 2A), yet lacked other Th2-associated (*Ccr3, Ccr8, Il4, Il5, Il9*) or Tfh-associated (*Cxcr5, Cxcl13, Il21*) genes (Figure S1B), suggesting that nfc1 does not align with either Th2 or Tfh lineage. Th9-related (*Spi1*, *Il4ra*, *Il9*) and Th22-related (*Ahr*, *Ccr4*, *Ccl7, Il13*, *Il22*) genes were not observed either (Figure S1B), so these two lineages have no similarity with the nfc clusters. Interestingly, the nfc1 and a part of the nfc2 populations shared several T cell markers with naïve or resting cells (*Ccr7*, *Lef1*, and *Tcf7*) ^25^ (Fig. 2C).

We next considered Foxp3-negative regulatory phenotypes. Type 1 T-regulatory (Tr1) cells are featured as IL-10 producers independent of Foxp3^26^, but the nfc cells showed little to no *Il10* expression nor IL-10 production (Fig. 2A–B and Fig. 2D), and this would argue against them being Tr1 cells. Instead, both nfc clusters showed high expression of *Tgfb1* (Fig. 2A–B), consistent with a Th3 phenotype involved in mucosal immune regulation and oral tolerance ^27^. While they expressed LAP-1 (latency-associated peptide, a product of *Tgfb1*), unlike some gut Tregs, they did not express IL-10 ^28^ (Fig. 2D). Of note, *Tgfb2, Tgfb3* and the immunosuppressive cytokine IL-35 (*Il12a* and *Ebi3*) were undetectable in all eye-primed clusters (Figure S1C).

These data support the conclusion that the nfc clusters are Th-lineage-negative, and as such, are unlikely to represent known pathogenic effector cells or canonical Tregs.

### The nfc clusters exhibit a combination of regulatory/anergic gene signature

**Fig. 3:**
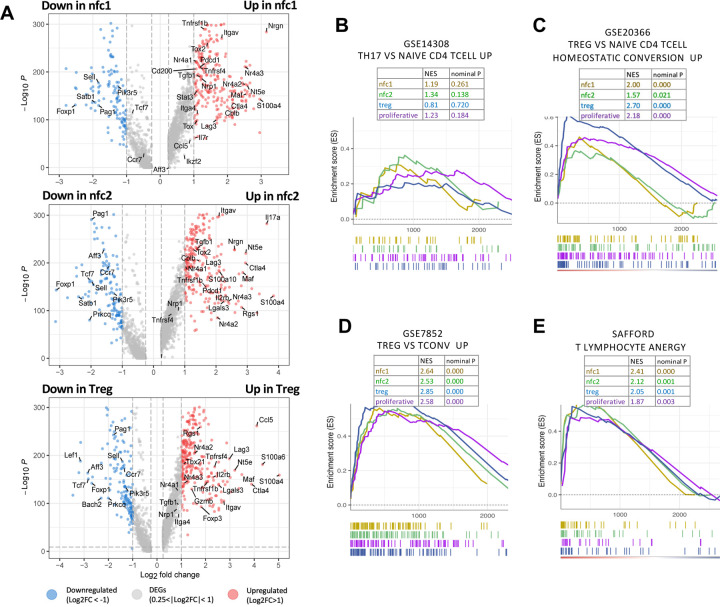
Intraocular environment induces regulatory and anergy signatures (A) Volcano plots showing the differential gene expression of each cluster (baseline: naive cluster). Differentially expressed genes with more than 2-fold changes are highlighted in red (upregulated) or blue (downregulated). Representative signature genes are shown. (B-E) Representative GSEA enrichment results mapping the signatures of each cluster against the Molecular Signature Database (MSigDB). NES, normalized enrichment score, indicating the similarity of the current gene signatures with predefined gene sets. Nominal P value lower than 0.05 denotes significant similarity to the corresponding to the predefined gene set in B, C, D or E. (B) Signature of Th17 cells. (C) Signature of de novo converted Treg cells in vivo (also known as peripherally induced Tregs). (D) Signature of in vivo Treg cells in lymphoid tissues (spleen, thymus, and lymph nodes). (E) Curated anergy signature from canonical in vitro anergy-inducing conditions

To better characterize the nfc clusters, we then investigated their global transcription profile. Gene signatures of each subset in the eye were defined by comparing their transcriptomes to that of naive T cells as baseline (Fig. 3A and Table S2). Both nfc clusters expressed high levels of anergy-associated genes as *Nrgn* ^29 30^, *Cblb* ^31^, *Dgkz* ^32^, *Nr4a1–3* ^33,34^, *Nrp1*, *Tox*, and *Tox2* ^16,35,36^. They also expressed canonical Treg-related genes, including inhibitory checkpoint molecules (*Nt5e*, *Maf*, *Itgav*, *Il2rb*, *Tnfrsf4*, *Tgfb1, Ctla4*, *Lag3*) ^37–41^ (Fig. 3A), and these were shared with the Treg cluster. Of note, *Ccl5*, *S100a4*, *S100a6*, *Tbx21*, and *Gzmb,* which characterize activated, highly suppressive Tregs ^38–40^ and were present in the Treg cluster, were not shared with the nfc populations (Fig. 3A).

We then performed Gene Set Enrichment Analysis (GSEA) to align the signatures of eye-primed T cell clusters with predefined gene sets in the Molecular Signature Database (MSigDB) ^42^ (Fig. 3B–E). In spite of upregulated *Il17a* (Fig. 3A, middle), the nfc2 cluster did not have a characteristic Th17 signature ^43^ (Fig. 3B). Rather, the two nfc clusters appeared to share characteristics with Treg cells that had been *de novo* differentiated in non-ocular tissues *in vivo* ^37^ (Fig. 3C), Tregs isolated from lymphoid tissues of healthy mice ^44^ (Fig. 3D) and to *in vitro* induced anergic T cells ^45^ (Fig. 3E). The Treg cluster also shared genes with the anergic signature (Fig. 3E), supporting the notion that many phenotypic and mechanistic traits are shared between Treg and anergic T cells defined by other studies ^46–48^. Given that the nfc clusters lacked the defining Treg gene Foxp3, in the aggregate, they conformed best to the ‘T lymphocyte anergy’ gene set.

### The nfc clusters are hyporesponsive to antigenic stimulation but lack suppressive function

**Fig. 4:**
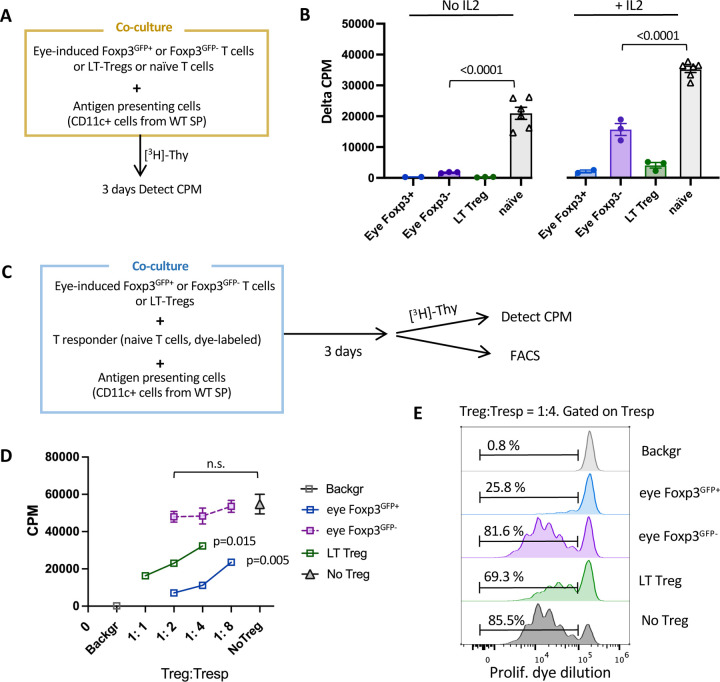
Non-Foxp3-converted (nfc) cells are functionally hyporesponsive to cognate antigen but not suppressive (A) Diagram of co-culture system in B. Naïve T cells or Tregs from peripheral lymphoid tissues (LT) were sorted from Tcra−/− R161H Foxp3GFP mice. Donor-derived Tcra−/− R161H Foxp3GFP+ and Foxp3GFP– cells were sorted from recipients’ eyes. Antigen presenting CD11c+ cells were magnetically enriched from the spleen of WT B10.RIII mice. (B) Proliferation to the cognate antigen human IRBP161–180 in the presence or absence of IL-2. Cells without antigen served as background. Only one or two wells of eye-induced Treg cells could be set up per experiment. Shown is one representative experiment of 3. (C) Scheme for antigen-specific suppression assay by [3H]-Thymidine uptake and dye dilution methods for D and E. (D) Dose-dependent suppression of T responder cells by putative suppressors with antigen stimulation (triplicates). One representative experiment of two. (E) FACS plots of proliferation dye dilution. Tresp without antigen served as background.

The results above suggested that the non-Foxp3-converted clusters were more reminiscent of Treg cells than of other Th cell lineages, and strongly resembled anergic T cells. Previous studies had demonstrated that a functional characteristic of anergy is hyporesponsiveness to antigenic stimulation, which can be rescued by IL-2 ^49^. To examine whether this was true of our nfc populations, we separated Foxp3+ and Foxp3– cells retrieved from the eye by flow sorting. Tregs and naïve T cells from peripheral lymphoid tissues (LT) of *Tcra*^−/−^ R161H Foxp3^GFP^ transgenic mice were used for comparison. Proliferation was measured by [^3^H]-Thymidine incorporation in a co-culture system with antigen presenting cells (APC) and Interphotoreceptor Retinoid-Binding Protein (IRBP) peptide as cognate antigen (Ag) (Fig. 4A). Eye-induced non-Foxp3-converted cells exhibited minimal proliferation, which was considerably enhanced in the presence of IL-2, although it remained markedly lower than that of the naïve cells (Fig. 4B). As expected ^50^, Treg cells also responded to IL-2 supplementation. Together with Fig 3, we interpret our data to mean that (a) the phenotype of the non-Foxp3 converted T cells is consistent with anergy, and (b) these cells remain hyporesponsive to their cognate antigen independently of continued presence of Foxp3^+^ Tregs and of the ocular environment.

Hyporesponsiveness to antigen that can be rescued by IL-2 is consistent with anergy, but is not necessarily a distinguishing attribute from Tregs. To resolve this, we compared the ability of eye-induced Treg and non-Foxp3-converted (Foxp3^GFP**–**^) T cells to suppress proliferation of naïve responders (Tresp) to their cognate Ag (IRBP peptide presented on splenic APC, Fig. 4C). We used two complementary methods: [^3^H]-Thymidine uptake and proliferation dye dilution, to exclude interference from possible proliferation of the Tregs themselves. Eye-induced Tregs and LT Tregs suppressed Thymidine uptake by Tresp in a dose-dependent fashion. In contrast, the eye Foxp3^GFP**–**^ cells failed to significantly inhibit Tresp proliferation even at a 1:2 Treg:Tresp ratio (Fig. 4D). Dye dilution analysis confirmed that the proliferation rate of Tresp in the presence of anergic cells did not differ from control, indicating that Foxp3^GFP**–**^ cells lacked appreciable suppressor function *in vitro* (Fig. 4E).

Therefore, from here on, the non-Foxp3-converted nfc1 and nfc2 clusters will be referred to as ‘anergic1’ and ‘anergic2’, respectively.

### Distinct genes contribute to dampened TCR signaling in eye-induced anergic and regulatory cells.

**Fig. 5:**
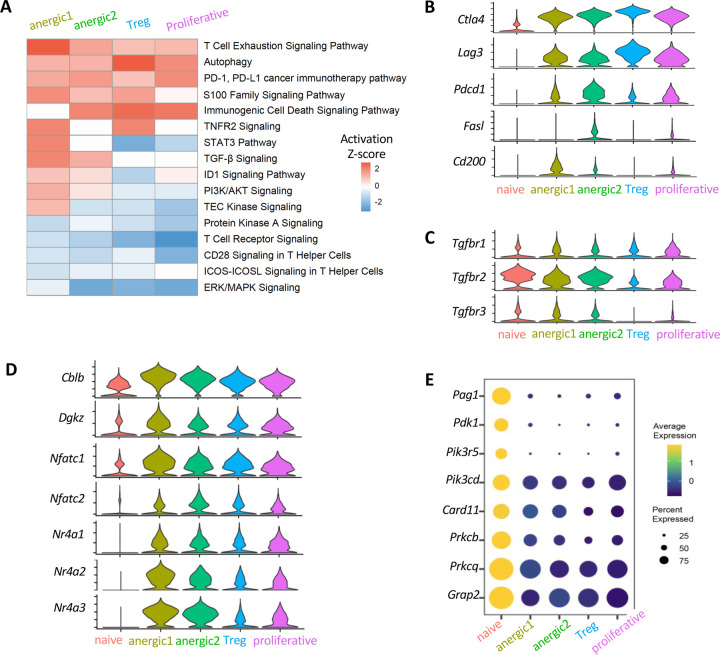
Suboptimal TCR signaling and inhibitory signals are involved in eye-induced tolerance (A) Heatmap of differentially regulated immune-related signaling pathways in each T cell population compared to the naive baseline. Z score was calculated by Ingenuity Pathway Analysis (IPA). (B-C) Violin plots showing expression of canonical inhibitory markers (B), and TGF-β receptors (C). (D) Violin plots displaying gene expression of anergy-associated factors. (E) Bubble plot showing frequency of positive cells (size of bubble) and expression levels (color gradient) of genes involved in TCR/CD28 and MAPK signaling pathways..

To address the question of what signaling molecules and pathways were involved in the induction the anergic and regulatory phenotypes, we performed an Ingenuity Pathway Analysis (IPA). Compared to the naïve cluster, pathways related to T cell suppression (T cell exhaustion, immunogenic cell death and TNFR2 signals) and deficient T cell receptor (TCR) signaling (downregulated T cell receptor, CD28, and ICOS signals) that restrain effector differentiation, were prominent in both Treg and anergic T cell clusters (Fig. 5A). Co-inhibitory genes that may feed into this pattern *Ctla4* and *Lag3* ^51,52^ were significantly upregulated in all eye-primed cell clusters, and their expression was highest in the Treg cluster, whereas *Pdcd1* (encoding PD-1), *Fasl* and *Cd200* were mainly increased in anergic clusters (Fig. 5B). Activation of TGF-β signaling in the anergic clusters (Fig. 5A) aligns with increased expression of TGF-β receptors (*Tgbr1–3*) (Fig. 5C), suggesting a connection between TGF-β signaling and the anergic state.

Compared to Tregs, the anergic populations expressed higher levels of anergy-inducing factors such as NFAT (nuclear factor of activated T cells, encoded by *Nfatc1/2*) family members ^53 54^, NR4A (nuclear receptor subfamily 4A, encoded by *Nr4a1–3*) family members ^33,34^, CBL-B ^31^, and DGKζ ^32^, which are causally related to hyporesponsiveness, by affecting multiple components participating in TCR signal transduction ^16,55^ (Fig. 5D). This is consistent with the low expression of molecules downstream of TCR/CD28 pathways, such as pyruvate dehydrogenase kinase 1 (*Pdk1)*, PI3Ks (*Pik3r5, Pik3cd*), and PKCs (*Prkcb, Prkcq*) (Fig. 5E), supporting dampened TCR/CD28 signaling.

### Distinct as well as shared tolerance-inducing regulons are enriched in anergic and Treg clusters

**Fig. 6:**
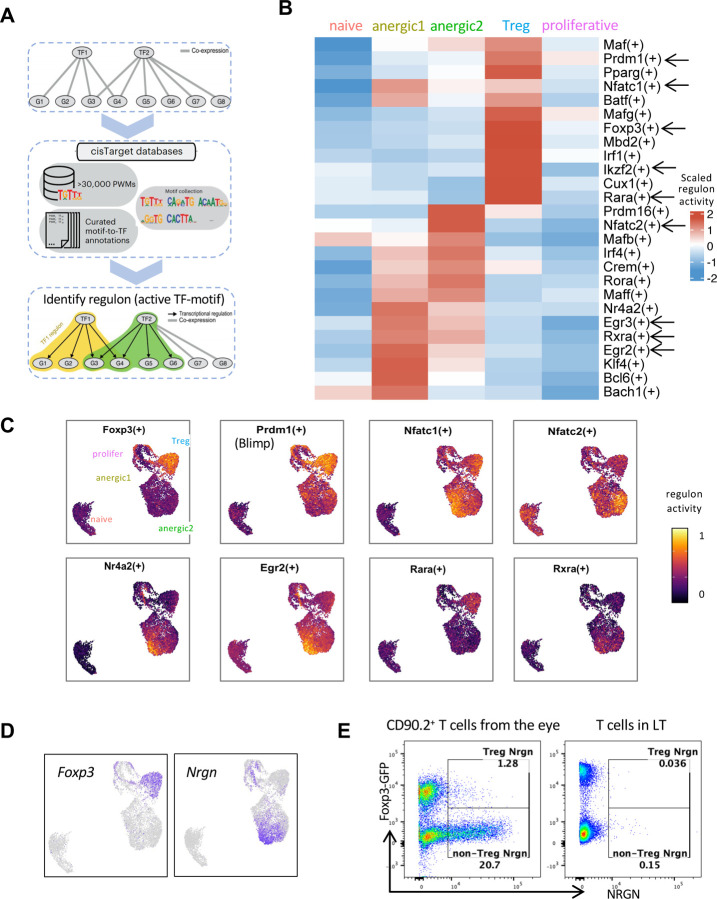
Distinct tolerance-inducing regulons and markers identify anergic and Treg cells (A) Workflow of regulon identification by SCENIC (single-cell regulatory network inference and clustering) analysis. (B) Representative regulons enriched in eye-primed T cell subpopulations and naive T cells are marked with arrows in the heatmap. (C) Visualization of the regulon activity overlaid on the UMAP. (D and E) Reciprocal expression of Foxp3 and Nrgn in Treg and non-Treg (anergic) clusters by RNA (D) and protein (E).

To identify potential key transcription factors (TF) regulating anergic and Treg subsets, we used the SCENIC (Single-Cell rEgulatory Network Inference and Clustering) pipeline, which infers TF activity from the co-expression of its direct target genes, collectively forming a regulon ^56^ (Fig. 6A). This provides a more precise readout of a cell’s state of differentiation than the TF mRNA expression alone.

Each of the anergic T clusters exhibited enrichment of distinct regulon activity. However, both clusters showed enrichment of regulons *Egr2*, *Egr3* ^45^, *Nr4a2,* and *Klf4* ^57^ (Fig. 6B). These TFs have been implicated in attenuating pathogenic T cell responses ^33^. Egr-2 in particular is considered a ‘master TF’ of anergy that directly upregulates CBL-B, DGKζ and other anergy-associated genes ^31,45,58^.

In the Treg cluster, Foxp3 and its ‘accessory’ TFs *Prdm1* (encoding BlimpT), *Ikzf2* (Helios) and *Mbd2* (Mbd2), that are known to promote and stabilize Treg function ^59–62^ exhibited increased regulon activities ^59^ (Fig. 6B). Enrichment of those Foxp3 accessory regulons is compatible with the high suppressive function of the eye-induced Treg cells that we observed (Fig. 4D). A total of 255 regulons were identified as active within our dataset (Table S3). Of note, regulons corresponding to T-bet and RORγt were not enriched, confirming that eye-primed populations did not appear to be Th1 or Th17 effectors.

Regulons active in both anergic and Treg clusters were *Nfatc1* and *Nfatc2* (Fig. 6C), which aligns with the known tolerogenic role of the NFAT family in both anergic cells and Tregs. In the former, they cooperate with NR4A and EGR2/3, to repress effector cytokines (IFN-γ, GM-CSF) and to induce inhibitory regulators CTLA-4, PD-1, LAG3, CBL-B and DGKζ ^58,63^, and in the latter NFAT-Foxp3 interaction upregulates Treg markers CTLA-4 and CD25, contributing to suppressor function ^54,64^. Moreover, we noticed the presence of regulons for the nuclear receptors for retinoic acid, *Rara and Rxra*. Of note, *Rara* regulon was highly activated in Tregs, whereas *Rxra* regulon was preferentially activated in anergic cells (Fig. 6B and 6C), suggesting that although RA regulates both Tregs and anergic cells, they rely on different receptors for RA mediated functions.

RARA is associated with Foxp3^+^ Treg development ^65^; however, since the anergic cluster lacks Foxp3, we looked for molecules downstream of the RXRA receptor. One gene known to be downstream of *Rxra*, *Nrgn* ^66^, was among the most highly expressed genes in the anergic clusters (Fig. 3A and Fig. 6D), and the top gene in the anergic1 cluster. Notably, its expression closely matched the distribution of *Rxra* regulon activity (Fig. 6C–D) and was mutually exclusive with Foxp3 expression (Fig. 6D). Control lymphoid tissue (LT) CD4^+^ cells, whether Foxp3^+^ or Foxp3^–^, lacked neurogranin (encoded by *Nrgn*) expression (Fig. 6E). *Nrgn* has normally been associated with neurons ^67^; here, we demonstrate high expression of *Nrgn* as well as its protein product, neurogranin, restricted to the eye-derived Foxp3-negative, i.e. anergic cells.

### Eye-induced Tregs and anergic cells seem to differentiate in parallel rather than sequentially

An important question in understanding the development of Treg and anergic cell fates is whether they differentiate as separate lineages or whether one derives from the other. To address this question, we performed trajectory analyses using RNA velocity ^68,69^ and Monocle pseudo-time trajectory analyses ^70^.

**Fig. 7:**
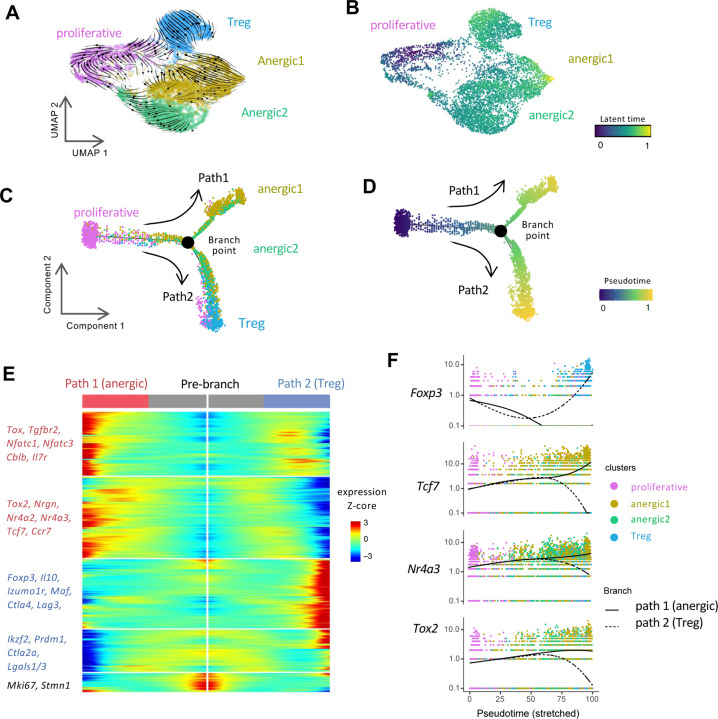
Trajectory analyses indicate parallel differentiation of Treg and anergic populations from an initial proliferative precursor. (A-B) RNA velocity analysis. Eye-primed cells were extracted and re-clustered for a new UMAP. (A) RNA velocity (arrowheads) projected onto this UMAP reflect the direction of cell state transitions. (B) The latent time is the calculated progression from the origin (proliferative cluster) to the end states (anergic1 and Treg), represented by color code. (C-F) Monocle pseudotime analysis. Monocle analysis ordered the cells along the pseudotime trajectories, displaying a branched pattern of two paths. (C) Color represents each cluster. (D) Color represents pseudotime, from the initial phase (0) to the late stage (1). (E) Heatmap showing the bifurcation of gene expression dynamics along pseudotime. (F) Kinetic patterns of specific canonical genes of the two paths. Cells were color-coded for each cluster.

RNA velocity can infer the direction of cellular state changes and estimate the future state based on the relative abundance of spliced and un-spliced transcripts ^68^. After excluding the naïve population and reclustering the eye-primed cells, we projected the RNA velocity vectors onto a new UMAP (Fig. 7A). The trajectories originated from the proliferative cell cluster and diverged in distinct directions. The Treg and anergic1 cluster appeared to be independent fates, with the arrows pointing in opposite directions, while a part of anergic2 population appeared to transition toward the anergic1 stage (Fig. 7A). Based on the estimated latent time by the scVelo algorithm ^69^, which reflects the internal clock of a cell (Fig. 7B), the anergic2 cluster seems to represent a less differentiated state with lower latent time, whereas the Treg and anergic1 populations may be more terminally differentiated.

As a complementary approach, we reconstructed the trajectories using the Monocle algorithm ^70^. The inferred state of cell transition revealed the emergence of two branches, arising from the common proliferative population and bifurcating at the branch point (Fig. 7C). Notably, one path was populated mainly by anergic1 cells, and the other path was dominantly occupied by the Treg cells. The distribution of anergic2 cells in both branches may point to the plasticity of this cluster (Fig. 7C). The branched pseudotime results supported that the anergic1 and Treg clusters had reached their final stages (Fig. 7D).

Fig. 7E and Table S4 depict the genes undergoing the most pronounced dynamic changes when progressing along each of the two branched paths. Resting/quiescent state markers (*Ccr7, Il7r, Tcf7*) and anergy-associated genes (*Nfatc1/3, Nr4a2/3, Tox, Tox2, Cblb*) were progressively upregulated along the anergic path (Fig. 7E), suggesting that these cells gradually lost their effector potential. Conversely, higher levels of canonical Treg-associated genes (*Foxp3, Il10, Ikzf2, Prdm1*) along the Treg path, was consistent with progressive differentiation of Tregs (Fig. 7E). Furthermore, while representative anergy-associated genes, such as *Tcf7, Nr4a3,* and *Tox2*, progressively increased in the anergic path, they progressively decreased in the Treg path (Fig. 7F), recapitulating the dynamic acquisition of the respective phenotypes.

In summary, the trajectory analysis reveals a branched pattern in which naïve T cells primed within the eye differentiate largely in parallel, rather than in tandem, into Tregs and anergic T cells from a common proliferative precursor.

## DISCUSSION

We provide the first study that resolves at the single cell level how the living eye actively “disarms” the pathogenic potential of retina-specific T cells *in vivo*. Within the eye, incoming T cells encounter high levels of TGF-β (mainly the TGF-β2 isoform) ^71^. Retinoic acid (RA) is also abundant in the eye, owing to its function in the visual cycle. This creates a unique environment that has a central role in ocular immune privilege ^18^. Outside the eye, RA is made by CD130^+^ DC in the gut, where it enhances Treg differentiation and may contribute to food tolerance ^72^. Within the eye, in addition to the Foxp3^+^ Treg fate adopted by a minority of the naïve T cells, we show that the remaining majority adopts a phenotype consistent with anergy. This finding fills a major gap in understanding that was left by our previous data ^18^, which found a dampened expression of effector cytokines and TFs at the population level, but could not distinguish effectors being kept in check by Foxp3^+^ Tregs, from an alternative cell fate(s), nor could it resolve possible subset(s). Our current data dissect this in detail at the molecular level, and resolve the cell fates and their differentiation trajectory.

### Anergy vs. Regulation: unique gene expression in ocular tolerance

The induction of anergic T cells in the living eye is a novel and little-explored aspect of ocular immune privilege. As mentioned in the Introduction, previous concepts of ocular immune privilege were based largely on *in vitro* studies with isolated cell populations or ocular fluids, and most of those studies dealt with induction of Tregs ^3 4 9^. Although one study suggested that interaction of T cells with RPE cells *in vitro* can result in anergy, for obvious reasons this does not reproduce the complexity of the actual intraocular environment ^9^. Moreover, the transcriptome of the affected T cells was not characterized. Our current study identifies many anergy-associated genes (*Ctla4, Lag3*, *Pdcd1, Cblb, Dgkz*), as well as activated anergy-promoting transcription factors (NFAT, EGR2/3, NR4A, TOX families), that are shared with other models of T cell anergy ^16,55 73^. However, we also identify multiple genes whose expression pattern appears characteristic to eye-induced anergy.

#### Prominent examples are:

- *Nrgn* (neurogranin), which was in our hands restricted to the anergic T cell population, as was *Egr-2*, a known *Nrgn* inducer ^29^. Nrgn is constitutively expressed in neuronal cells, where it regulates synaptic plasticity ^67^. While a few studies reported *Nrgn* mRNA in lymphocytes ^22 30 29^, its functional contribution remains to be unraveled. The role of Nrgn is to modulate intracellular Ca++ levels through its interaction with Calmodulin ^74^. Specifically, Nrgn sequesters Calmodulin by physically binding to it, and makes it unavailable for binding with Ca++. RA promotes *Nrgn* gene expression by upregulating RA receptors, particularly RXR, which binds to the RA response elements (RARE) in the *Nrgn* promoter ^66,75^. Nrgn in turn binds to and sequesters Calmodulin, lowering available free calcium ^74^. We hypothesize that as this process occurs in the T cells that are in the process of differentiation in the eye, RA-driven RAR/RXR upregulation increases Nrgn, sequestering Calmodulin and reducing intracellular calcium and inhibiting Calcineurin and NFAT ^53 76^. Because Treg differentiation and functional activation requires high Ca++ levels ^53^, these conditions should skew the balance of Tregs and anergic T cells towards anergy. We propose that Nrgn, by regulating intracellular Ca^++^ availability, acts as a key checkpoint in the choice of anergic vs. regulatory cell fate by newly primed T cells differentiating from a common precursor in the TGF-β and RA-rich ocular environment. The validation of this central hypothesis in the regulation of ocular immune privilege and T cell anergy is the subject of a separate ongoing study.
- Although anergic T cells are generally thought to lack cytokine expression, the ocular anergic cells expressed a high level of *Tgfb1. Tgfb1* was expressed also by ocular Tregs, and about 40% of both populations expressed the TGF-β protein. To our knowledge, *Tgfb1* expression had not been previously reported in any model of anergic T cells, and may be a distinguishing feature of eye-induced anergy. Nevertheless, judging by the functional data, its expression did not confer regulatory function on the ocular anergic T cells.
- An anergy-associated gene that was not significantly expressed in ocular anergic T cells is *Izumo1r* (encoding FR4), which, together with expression of *Nt5e* (encoding CD73) and absence of Foxp3, is considered a defining phenotype of anergic T cells, but its function in anergy has not been elucidated. Our data suggest that it may not have a functional role in eye-induced anergy, or its role is redundant with that of a gene(s) differentially expressed in eye-derived vs. other anergic cells, such as *Dgkz, Cblb, Rgs1, Maf, Lgals*7, and *Furin* ^55^.

### Eye-induced anergic state differs from exhaustion

Although T cell anergy and exhaustion share many transcriptional features, the development of the eye-induced anergic T cells is inconsistent with exhaustion for several reasons. Exhaustion occurs in environments with strong antigenic stimulation and efficient antigen presentation ^77^. The healthy eye has few and quiescent professional antigen-presenting cells (APCs) ^78–80^. Inefficient antigen presentation is conducive to T cell anergy induction rather than exhaustion. As well, exhaustion typically requires chronic Ag stimulation and follows full activation for effector function, whereas the cells here were analyzed after only one week of Ag exposure, and the retina had minimal pathology ^18^. Finally, many exhaustion-associated genes, such as *Tigit, Havcr2, Shp1–2, Ptpn2, Blimp1* and *Irf4* ^77 35^ were undetectable or minimally expressed in eye-induced anergic cells.

### Effector Treg characteristics define the ocular Treg Population

The gene expression profile of eye-induced Tregs (*Il10*, *Tgfb1*, *Ctla4*, *Lag3*, and *Nt5e*) is consistent with a highly suppressive “effector Treg” phenotype ^38–41^. This was confirmed functionally by comparison with Tregs from spleen and lymph node tissues of the same animals (note that all T cells are IRBP-specific). Regulon analysis also uncovered that many “Foxp3 accessory TFs”, such as Blimp1, Helios, and Mbd2, are activated. These TFs help maintain Treg stability ^59–62^, suggesting that Tregs differentiated within the eye may have a stable phenotype. Of interest, eye-induced Tregs also displayed some Th1-like genes, as indicated by higher levels of *Tbx21*, *Cxcr3*, and *Ccr5* compared to non-Foxp3 anergic cells, whereas the anergic2 population shared the lineage-specific marker *Il17a* with Th17 effector phenotype. Expression of lineage-specific genes shared with Th1 and Th17 effector cells by Tregs is felt to facilitate interaction with the target effector population(s) ^81–83^. Uveitogenic effector T cells are a mixture of Th1 and Th17 ^84^. It is therefore tempting to speculate that the ocular microenvironment diverts ‘would-be’ Th17 effectors to anergy, whereas ‘would-be’ Th1 effectors are diverted to Foxp3^+^ Treg fate. Investigation of this hypothesis and of the unique eye-induced Treg phenotype is part of a separate ongoing study.

### Limitations of the study

While the *in vivo* model of immune privilege is a powerful tool to dissect eye-specific control of immune cell differentiation, the system also has limitations, both objective and subjective. The level of complexity of an *in vivo* system precludes analysis of the individual contributions of signals from each component that integrate to produce the final phenotypic and molecular events. In part, this could be addressed by including the various ocular resident cells in the analysis. By the same token, RNA-Seq performed at additional time points could provide further insights into the kinetics of the differentiation process that could have strengthened our conclusions from the trajectory analysis. However, technical and logistic difficulties inherent to this experimental model precluded addressing this in the current study.

### In conclusion

Our findings shed new light on the concepts of ocular immune privilege and the molecular mechanisms that actively maintain immunological homeostasis. The results lead to a model in which the ocular environment limits pathology by instructing the conversion of conventional T cells to Tregs or to an alternative fate of T cell anergy, rather than a scenario where a population of Tregs keeps a population of T effector cells in check. Identification of eye-induced regulatory and anergic signatures offers a valuable foundation for future research, and may inform therapeutic strategies for ocular inflammatory diseases. Furthermore, these unique signatures may inform strategies to reverse undesirable T cell unresponsiveness in contexts such as cancer, vaccination and chronic infection.

## STAR METHODS

### MATERIALS AND METHODS

#### Mice

Interphotoreceptor retinoid-binding protein (IRBP)-specific T cell receptor (TCR) transgenic (R161H), *Tcra* knockout mice (*Tcra*^−/−^) on the B10.RIII background were generated as previously described ^85^ and were crossed to BTb.RIII Foxp3^GFP^ strain ^86^. *Tcra*^−/−^ R161H Foxp3^GFP^ reporter mice were used as donors for naive retina-specific T cells. CD90.1 congenic wildtype (WT) B10.RIII mice were used as recipients. Both male and female mice 6–10 weeks old were used in this study. All animals were maintained under specific-pathogen-free conditions at NIH animal facility on standard chow and water ad libitum. Care and use of animals followed institutionally approved animal study protocols and Animal Research Advisory Committee (ARAC) guidelines.

#### Ocular Immune Privilege Model

The ocular privilege model was established and described in our previous study ^18^. Briefly, retina-specific T cells, enriched from peripheral lymphoid tissues of *Tcra*^−/−^ R161H Foxp3^GFP^ donor mice using CD3^+^ T cell enrichment columns (R&D Systems) or CD4^+^ T cell isolation kit (Miltenyi Biotec), were FACS sorted to obtain the naive population depleted of preexisting Tregs (CD44^low^ CD25^-^ Foxp3^GFP-^). WT CD90.1-congenic recipient mice were injected intravitreally with 500,000 of these naïve T cells in 1.5 microliters PBS into each eye, using a 33G needle and Hamilton syringe. The cells were retrieved from donor eyes 7–8 days later and prepared for analysis, as described ahead.

#### Flow cytometry and cell sorting

Single-cell suspensions from spleens and lymph nodes (submandibular, axillary, inguinal, and mesenteric lymph nodes) collected from *Tcra*^−/−^ R161H Foxp3^GFP^ CD90.2 donor mice were used for isolation of retina-specific T cells. Cells were stained with surface antibodies and sorted for live CD4^+^ CD44^low^ Foxp3^GFP-^ CD25^-^ Dump^-^ cells to ~99% purity using FACSAria II and AriaIII/Fusion sorters (BD Biosciences). Non-CD4 markers (CD8, NK1.1, B220, CD11b, DX5, and Gr1) were used for the dump channel. To retrieve retina-specific T cells from the eyes of CD90.1 congenic recipients, eyes were minced and treated with T mg/ml collagenase D for 30 min at 37°C. Donor-derived retina-specific T cells were sorted as live CD4^+^ CD90.2^+^ CD90.1^-^ cells. Propidium Iodide or 7-AAD (for sorting) and ViaKrome 808 (for flow analysis on CytoFlex LX, Beckman Coulter) were used to exclude dead cells. For intracellular staining of cytokine, cells were stimulated with PMA (10 ng/ml) and ionomycin (500 ng/ml) in the presence of brefeldin A (GolgiPlug; BD) for 4 h, following staining for surface marker and live/dead cells. Cells were then fixed with 4% paraformaldehyde for 30 mins and stained for intracellular proteins in 1x BD perm/wash buffer for 1 hour. For Nrgn staining, cells were stained with surface maker antibodies and then fixed with 4% paraformaldehyde, followed by permeabilization and staining with Nrgn antibody at 4°C for 30 mins, and AF-647 conjugated anti-Rabbit secondary antibody for 20 mins. Antibodies used for cell sorting and flow cytometry analysis were from BD Biosciences, BioLegend, and eBioscience/ThermoFisher. Detailed antibody and clone information is listed in Table S1.

#### Sample processing and scRNA-seq

Fresh naive donor T cells, or donor cells retrieved from recipient mouse eyes one week after intravitreal injection, were collected for scRNA-seq. Donor T cells in the eyes retrieved from each of the four recipient mice were individually labeled with anti-mouse TotalSeqB hashtags (BioLegend, Table S1). Individual samples were incubated with unique hashtags and sorting antibodies before FACS sorting, per manufacturer’s protocol. The viability of sorted cells was greater than 90%. Sorted single-cell suspensions were adjusted to 700–1200 cells/μl before loading the 10X chromium chip. Samples were processed with the Chromium Next GEM Single Cell 3’ reagent kit in the Chromium X platform following the standard protocol for 3’ Gene Expression assay (TbX genomics). The gene expression and cell surface libraries were sequenced on NovaSeq 6000 platform (Illumina).

#### Quality control and clustering of scRNA-seq data

Sequencing reads were demultiplexed and aligned using CellRanger (7.1.0) with the default parameters. The output matrix files were converted into a Seurat object for quality control and clustering. Standard scRNA-seq analysis (quality control, clustering, and marker gene detection) was performed using Seurat (v4.3.0) ^21^. Cells were excluded from analysis if they met any one of the following criteria: transcript counts less than 1000 or more than 40000, fewer than 400 genes, more than 8% mitochondrial fraction, ribosomal fraction less than 10% or more than 45%. Highly variable features between individual cells were identified, and linear dimensional reduction was performed using principal component analysis (PCA). Unsupervised clusters were determined using the ‘FindNeighbors’ and ‘FindClusters’ functions based on the first 20 principal components (PCs). The clustering result was visualized using 2D uniform manifold approximation and projection (UMAP). Clustering was done at 0.2 resolution to keep the naive T cells as one “homogeneous” cluster. The ‘FindAllMarkers’ function was used to identify marker genes of each cluster within the data set.

#### Signature identification and GSEA analysis

To characterize the phenotypes of donor T cells retrieved from recipient eyes, we defined their gene-expression signatures and performed Gene Set Enrichment Analysis (GSEA). The gene signatures were defined by comparing each cluster with the naive cluster using the ‘FindMarkers’ function. Genes were considered differentially expressed using the default Wilcoxon Rank Sum test and log fold-change (FC) threshold (Table S2). The full list of differentially expressed genes (DEGs) was ranked based on log FC and then mapped to the Molecular Signature Database (MSigDB) via GSEA software (v4.3.2) ^42^.

#### Antigen-specific proliferation assay

Ocular immune privilege model was conducted as described above, after one week, Foxp3^GFP+^ or Foxp3^GFP-^ CD4^+^ CD90.2^+^ CD90.1^-^ cells were sorted out from the recipients’ eyes (50,000 cells/ well) and co-cultured with human IRBP_161–180_ peptide (50 ng/ml) and CD11c^+^ dendritic cells (at a 1:5 ratio to T cells), with or without 100 IU/ml recombinant human IL-2. Dendritic cells were obtained by digesting spleens from WT CD90.1 mice in spleen dissociation medium (Stem Cell) for 30 minutes, followed by ammonium-chloride-potassium (ACK) lysis and CD11c^+^ enrichment using Micro Beads (Miltenyi Biotec). Sorted naïve T cells (CD44^low^ CD25^-^ Foxp3^GFP-^ CD4^+^) and Treg cells (Foxp3^GFP+^ CD4^+^) from spleens and lymph nodes of *Tcra*^−/−^ R161H Foxp3^GFP^ CD90.2 mice were used as positive and negative controls, separately. Cell proliferation was determined using [^3^H]-Thymidine incorporation by adding 1mCi/well after a 48-hour culture and further incubated for 16 hours. Samples were harvested and counted using liquid scintillation (Perkin Elmer, MA). Unpaired Student *t*-tests were performed for statistics.

#### Antigen-specific Treg suppression assays

Sorted Foxp3^GFP+^ or Foxp3^GFP-^ CD4^+^ CD90.2^+^ CD90.1^-^ cells from recipients’ eyes were co-cultured with naïve retina-specific T cells (serve as T responder cells, Tresp; 50,000 cells/ well) and CD11c^+^ dendritic cells (at a 1:5 ratio to Tresp). Treg cells (Foxp3^GFP+^ CD4^+^) from spleens and lymph nodes of *Tcra*^−/−^ R161H Foxp3^GFP^ CD90.2 mice were used as positive control of suppressor. Varying numbers of putative suppressor T cell populations were sorted and added to the cultures at the indicated Treg:Tresp ratios. Cell co-cultures were stimulated with 50ng/ml human IRBP_161–180_ without adding IL-2. The inhibitory effect was assessed using either [^3^H]-Thymidine incorporation or proliferation dye dilution independently. For the dye dilution method, naïve T cells were labeled with proliferation dye - CellTracker DeepRed or CellTrace FarRed (Invitrogen/ThermoFisher Scientific) before setting up the culture. After 3 days, cells were stained for FACS analysis, and cell division of Tresp was quantified using FlowJo (10.8.0). Unpaired Student *t*-tests were performed for statistics.

#### Ingenuity Pathway Analysis

Pathway analysis was performed using Ingenuity Pathway Analysis (IPA, www.qiagen.com/ingenuity). DEGs of each eye-primed cluster with corresponding log FC and adjusted P values were imported into IPA software for deciphering upregulated or downregulated functional pathways based on ingenuity knowledge base. After performing ‘core analysis’ of each T cell cluster independently, visualization across different clusters was achieved by ‘comparison analysis’ function. IPA’s z-score indicates a predicted activation or inhibition of a pathway, where a positive z value denotes an overall pathway’s activation and vice versa.

#### The transcriptional factor activity (regulon) analysis

The python implementation of SCENIC (single-cell regulatory network inference and clustering, pySCENIC, v0.12.1) ^56^ was used to predict the active transcriptional factor (TF). Starting from the normalized matrix data, the pySCENIC workflow consists of three stages. Initially, co-expression modules were inferred using a regression per-target approach. Then, the regulons (TF-target gene motifs) were refined from these modules based on cisTarget databases. Lastly, the ‘aucell’ algorithm was utilized to quantify the regulon activity score and find the significantly enriched regulon independently for each cell with default parameters. Information on software tools and cisTarget databases can be found in Table S3. The statistically significant regulons identified by SCENIC analysis were considered as active TFs, which reflected the upstream transcriptional drivers of the observed cellular identities ^56^. Regulon activity score was then scaled to plot heatmap or projected onto the UMAP.

#### RNA velocity analysis

Velocyto ^68^ and scVelo ^69^ packages were used to perform RNA velocity analysis. First, RNA velocity (comprising spliced/un-spliced counts) for each cell was computed using the matrices generated by CellRanger and stored in the loom format. The velocity vectors were integrated into the Seurat object as a new data file. From the following file, we extracted the cells retrieved from recipient eyes and re-plotted the UMAP. The ‘latent time’ and ‘latent time facilitated RNA velocity’ were estimated using the likelihood-based dynamical model in scVelo. The velocity graph was visualized as streamlines overlaid by embedding in UMAP.

#### Pseudotime analysis

Monocle (v2.26.0) ^70^ was applied to determine the potential lineage differentiation trajectory, keeping the default parameters. The matrix data of eye-primed clusters were imported as input for creating the Monocle ‘CellDataSet’. The ‘DDRTree’ method was utilized for dimensionality reduction and cell ordering along the pseudotime trajectory. To identify the genes that separate cells into branches, we performed the Branch Expression Analysis Modeling (BEAM) approach in Monocle 2. The dynamic expression of genes was visualized by the ‘plot_genes_branched_heatmap’ or ‘plot_genes_branched_pseudotime’ function.

## Supplementary Material

Supplement 1

Supplement 2

Supplement 3

Supplement 4

Supplement 5

## Figures and Tables

**Table:** 

REAGENT or RESOURCE	SOURCE	IDENTIFIER
Antibodies		
Anti-mouse CD44	eBioscience/Thermofisher	103059
Anti-mouse CD25 (IL2R)	BD Biosciences	47-0251-82
Anti-mouse CD90.1	BioLegend	109006
Anti-mouse CD90.2	BioLegend	140312
Anti-mouse CD4	BioLegend	116025
Anti-mouse CD8a	BioLegend	162306
Anti-mouse NK1.1	BD Biosciences	550627
Anti-mouse CD45R (B220)	BD Biosciences	553092
Anti-mouse CD11b	BioLegend	101212
Anti-mouse CD49b (DX5)	BD Biosciences	558295
Anti-mouse Gr1 (Ly6C/Ly6G)	BioLegend	108412
Anti-mouse Neurogranin	Invitrogen/ Thermofisher	PA5-76392
Anti-mouse IL-10	BD Biosciences	53-7101-82
Anti-mouse LAP-1	BD Biosciences	141404
Goat anti-Rabbit secondary Antibody AF-647	Invitrogen/Thermofisher	A-21245
Fc Block	BD Biosciences	553141
Trustain FcX PLUS (anti-mouse CD16/32)	BioLegend	156603
TotalSeq^™^-B anti-mouse Hashtag (#1 - #4) antibodies	BioLegend	155831, 155833, 155835, 155837
Chemicals, peptides, and recombinant proteins		
Mouse CD3^+^ T cell enrichment columns	R&D Systems	MTCC-10
CD4^+^ T cell isolation kit	Miltenyi Biotec	130-104-454
CD11c^+^ enrichment using Micro Beads	Miltenyi Biotec	130-125-835
MS Columns	Miltenyi Biotec	130-042-201
LS Columns	Miltenyi Biotec	130-042-401
Human IL-2 (Proleukin)	Novartis	BT-002-100
Collagenase D	Roche	45-11088866001
Propidium Iodide	Millipore Sigma	25535-16-4
7-AAD	eBioscience/Thermofisher	00-6993-50
ViaKrome 808	Beckman Coulter	C36628
IRBP_161-180_ peptide (SGIPYIISYLHPGNTILHVD)	Bio Basic Inc.	Custom synthesis
^3^H Thymidine	Perkin Elmer Health Sciences, Inc	NET027A005MC
CellTracker Deep Red	ThermoFisher Scientific	C34565
CellTrace FarRed	ThermoFisher Scientific	C34564
SPRIselect Reagent kit	Beckman Coulter	B23318
Critical commercial assays		
Chromium Next GEM Single Cell 3’ Reagent kit v3.1	10X Genomics	PN-1000121
Chromium Single Cell 3’ Feature Barcode Library kit	10X Genomics	PN-1000079
Chromium Next GEM Chip G Single Cell Kit	10X Genomics	PN-1000127
Single Index Kit T Set A	10X Genomics	PN-1000213
Deposited data		
Raw and analyzed data	This paper and Gene Expression Omnibus	GEO: GSE281410
R code for analysis	Submitted to Github	https://github.com/NIH-NEI/Privilege_Treg_Anergy_scRNA
Experimental models: Organisms/strains		
*Tcra*^−/−^ R161H Foxp3^GFP^ CD90.2 mice	This paper	N/A
CD90.1 congenic B10.RIII mice	In house bred	N/A
Software and algorithms		
Cell Ranger (7.1.0)	10X Genomics	https://www.10xgenomics.com/support/software/cell-ranger/latest
Seurat R package (v4.3.0)	https://doi.org/10.1016/j.cell.2021.04.048	https://satijalab.org/seurat/
R package	R development core team	http://www.R-project.org
Gene Set Enrichment Analysis (GSEA) (v4.3.2)	https://doi.org/10.1073/pnas.0506580102	https://www.gsea-msigdb.org/gsea/index.jsp
Molecular Signature Database (MSigDB)	https://doi.org/10.1016/j.cels.2015.12.004	https://www.gsea-msigdb.org/gsea/msigdb
pySCENIC v0.12.1	https://doi.org/10.1038/s41596-020-0336-2	https://pypi.org/project/pyscenic/
Velocyto	https://doi.org/10.1038/s41586-018-0414-6	http://velocyto.org
scVelo	https://doi.org/10.1038/s41587-020-0591-3	https://scvelo.org
Monocle v2.26.0 (R package)	https://doi.org/10.1038/nmeth.4150	https://www.bioconductor.org/packages/release/bioc/html/monocle.html
Flowjo 10	BD Bioscience	https://www.flowjo.com/solutions/flowjo/downloads
GraphPad prism 10	GraphPad by Dotmatics	https://www.graphpad.com/resources
Ingenuity Pathway Analysis	Qiagen	www.qiagen.com/ingenuity
Python 3	Python.org	https://www.python.org/download/releases/3.0/
Other		
Hamilton Syringe 62RN	Hamilton company	7632-01
33G Hamilton Needle (0.375” Point style 4, 60° bevel angle)	Hamilton company	7803-05 Custom modified
FACS Aria II	BD Bioscience	N/A
FACS Aria III/FUSION	BD Bioscience	N/A
CytoFlex LX	Beckman Coulter	N/A
Chromium X	10X Genomics	1000331
NovaSeq 6000	Illumina	N/A
Scintillation counter	Perkin Elmer	N/A

## Data Availability

The data reported in this paper are deposited in the Gene Expression Omnibus (GEO) database under accession no. GSE281410. Code used for analysis can be found in GitHub https://github.com/NIH-NEI/Privilege_Treg_Anergy_scRNA.

## Highlights:

- Eye-primed retina-specific T cells develop distinct tolerance-associated phenotypes.
- Eye-induced anergic T cells remain hyporesponsive to antigen re-stimulation.
- Regulatory and anergic T cells differentiate in parallel from a common precursor.
- Induction of T cell anergy is a novel feature of ocular immune privilege.

## Abbreviations:

Ag: Antigen
AH: Aqueous humor
APC: Antigen presenting cells
CBL-B: Casitas B-Lineage Lymphoma Proto-Oncogene B
CTLA-4: Cytotoxic T-Lymphocyte Associated Protein 4
DEG: Differentially expressed genes
DGKζ: Diglyceride Kinase Zeta
EGR: Early Growth Response
FACS: Fluorescence-activated Cell Sorting
FC: Fold change
Foxp3: Forkhead box P3
GFP: Green fluorescent protein
GM-CSF: Granulocyte-Macrophage Colony-Stimulating Factor
GSEA: Gene Set Enrichment Analysis
ICOS: Inducible T-Cell Co-Stimulator
IFNγ: Interferon Gamma
IL-17A: Interleukin 17A
IPA: Ingenuity Pathway Analysis
IRBP: Interphotoreceptor Retinoid-Binding Protein
LAP: Latency-Associated Peptide
LT: Lymphoid tissues
MAPK: Mitogen activated protein kinase
MsigDB: Molecular Signature Database
NFAT: Nuclear factor of activated T cells
Nrgn: Neurogranin
NR4A: Nuclear receptor subfamily 4A
PC: Principal component
PD-1: Programmed Cell Death 1
PD-L1: Programmed Cell Death 1 Ligand 1
PI3K: Phosphatidylinositol-4,5-Bisphosphate 3-Kinase
PKC: Protein Kinase C
RA: Retinoic acid
RARA: Retinoic acid receptor alpha
RARE: Retinoic acid response elements
RXRA: Retinoid X receptor alpha
RPE: Retinal pigment epithelium
RORγt: Retinoid orphan receptor gamma t
SCENIC: Single-cell regulatory network inference and clustering
scRNA-seq: Single-cell RNA sequencing
TCR: T cell receptor
Th: T helper
TF: Transcription factor
Tfh: T follicular helper
TGF-β: Transforming growth factor-beta
TNFR2: Tumor Necrosis Factor Receptor 2
TOX: Thymocyte Selection-Associated High Mobility Group Box
Tr1: Type1 regulatory cells
UMAP: Uniform manifold approximation and projection
VIP: Vasoactive intestinal peptide
WT: Wild type
α-MSH: α-melanocyte-stimulating hormone
